# Supplementary material for: Extreme Low Cytosolic pH Is a Signal for Cell Survival in Acid Stressed Yeast
Source: Genes (Basel). 2020 Jun 16;11(6):656. doi: 10.3390/genes11060656 (PMC7349538; doi:10.3390/genes11060656)
Supplement: Supplementary file 1 [file genes-11-00656-s001.pdf]

De Lucena RM et al.

Extreme low cytosolic pH is a signal for cell survival in acid stressed yeast

Supplementary material

Table S1. List of peptides identified by mass spectrometry from the supernatant of wild type BY4741 strains (WT) and its isogenic mutant *slt2*Δ cultivated in YNB medium adjusted to pH 5.0 or pH 2.5 with sulphuric acid. The average number of peptides identified for a protein in two biological replicates are listed for each condition.

Name	Description	Subcellular localization[a]	# of peptides identified			
			WT 5.0	WT 2.5	*slt2*Δ 5.0	*slt2*Δ 2.5
Ygp1	Cell wall-related secretory glycoprotein	S	59.5	54.5	56.5	33.5
Gas1	1,3-β-glucanosyltransferase	GPI-PM[b]	61	58.5	48	16
Bar1	Aspartyl protease that inactivates alpha factor	S	53	20	45	5.5
Pry1	Involved in the export of acetylated sterols	CW/S	48.5	18	48	5.5
Zps1	Zinc- and pH-regulated surface protein	GPI[b]	44	13	52	9
Ecm33	GPI-anchored protein in cell wall biogenesis	GPI-CW[b]	36.5	21	31	6
Cts1	Endochitinase required for cell separation	CW (NC)[c]	32.5	13	25	2.5
Pst1	GPI-anchored protein in cell wall biogenesis	GPI-CW[b]	36	53	35	2
Bgl2	Cell wall endoglucanase	CW (NC)[d]	30.5	19.5	19	5
Gas5	1,3-β-glucanosyltransferase	GPI-PM[b]	21	16.5	18	4
Ccw14	Covalently linked cell wall protein	CW (C)[c]	10.5	13	7	5.5
Wsc4	Cell wall integrity and stress response	PM	10.5	7	13	1
Pho3	Constitutive acid phosphatase	S	8.5	5	10	3
Msb2	Mucin involved in various signaling pathways	PM	7	1.5	4.5	1.5
Sim1	Probable secreted β-glucosidase	CW (NC)[e]	395	10.5	32.5	
Scw4	Cell wall protein with similarity to glucanases	CW (NC)[c]	93.5	40	57.5	
Exg1	Major exo-1,3-beta-glucanase of the cell wall	CW (NC)[c]	48	29.5	40.5	
Egt2	Cell wall endoglucanase	GPI-CW[b]	45,5	5	36	
Uth1	Probable secreted β-glucosidase	CW (NC)[e]	38.5	8.5	22.5	
Crh1	Chitin transglycosylase	GPI-CW[b]	41	37.5	32	
Tos1	Cell wall protein of unknown function	CW (C)	30.5	9,5	22	
Scw11	Cell wall protein with similarity to glucanases	CW (NC)[d]	26	6	15	
Scw10	Cell wall protein with similarity to glucanases	CW (NC)[d]	26	42	17	
Pry2	Involved in the export of acetylated sterols	CW/S	18.5	17	13.5	
Nca3	Beta-glucosidase-like protein	S[e]	13.5	21.5	16.5	
Cis3	Cell wall mannoprotein	CW-PIR	18	19.5	23.5	
Cwp1	Cell wall mannoprotein	GPI-CW[b]	9.5	50	2	
Pir1	Cell wall mannoprotein	CW-PIR[c]	11	12	14	
Gas3	Putative 1,3-β-glucanosyltransferase	GPI-PM$_b$	7	3	3.5	
Pry3	Involved in export of acetylated sterols	GPI-CW[b]	5	3	4	
Yps3	Yapsin aspartic protease	GPI-PM[b]	3	37	1	
Kre9	Involved in cell wall β-glucan assembly	CW/S	24.5		19	
Vel1	Protein of unknown function	C/periphery	12.5		8.5	
Aim41	Involved in mitochondrial inheritance	M	14		16	

Dse4	Daughter specific protein, similarity to glucanases	CW/S	6.5		5.5	
Utr2	Chitin transglycosylase	GPI-CW[b]	6.5		5	
Hsp150	O-mannosylated heat shock protein	CW-PIR[b]	11		165	
Cwp2	Cell wall mannoprotein	GPI-CW[b]	3.5		2.5	
Ccw12	Cell wall mannoprotein	CW (C)[d]	4		2	
Pir3	Cell wall mannoprotein	CW-PIR[d]	8			
Hor7	Unknown function	GPI[b]	2.5			
Exg2	Exo-1,3-β-glucanase	GPI-PM[b]	1.5			
Toh1	Cell wall protein	GPI-CW[b]	1.5			
Yel073c	Unknown function	?	1			
Hsp12	Membrane organization during stress	C/PM		40.5	1	118.5
Zeo1	Cell wall stress signaling	PM		33	7.5	60.5
Tdh3	GAPDH, secreted for antimicrobial peptides	C/S[f]		3	12.5	40.5
Sun4	Probable secreted β-glucosidase	CW (NC)[e]		2	2	
Trx2	Thioredoxin isoenzyme	C		4,5		9
Plb1	Phospholipase B	GPI-CW[b]		3,5		
Eno2	Enolase	C			8,5	34,5
Pgk1	3-phosphoglycerate kinase	C			8	9
Tdh1	GAPDH	C/S[f]			7,5	16,5
Fba1	Fructose 1,6-bisphosphate aldolase	C			5,5	9
Pdc1	Major of three pyruvate decarboxylase isozymes	C			3,5	24,5
Cpr1	Peptidyl-prolyl cis-trans isomerase	N/C			2,5	1,5
Pgi1	Phosphoglucose isomerase	C			2,5	1

[a] CW, Cell wall (GPI-CW, coupled via GPI moiety to β-1,6-glucan; CW-PIR, coupled via alkali-sensitive β-1,3-glucan linkage; CW (C), covalently linked to wall via unknown linkage; CW (NC), non-covalently associated with cell wall); PM, Plasma membrane (GPI-PM, GPI anchored); S, Secreted; C, Cytoplasm; M, Mitochondria; N, nucleus

[b]de Groot *et al.*, 2003

[c]Cappellaro *et al.*, 1998

[d]Mrsa *et al.*, 1997

[e]Kuznetsov *et al.*, 2013

[f]Branco *et al.*, 2014
